# Microplastics Derived from Food Packaging Waste—Their Origin and Health Risks

**DOI:** 10.3390/ma16020674

**Published:** 2023-01-10

**Authors:** Kornelia Kadac-Czapska, Eliza Knez, Magdalena Gierszewska, Ewa Olewnik-Kruszkowska, Małgorzata Grembecka

**Affiliations:** 1 Department of Bromatology, Faculty of Pharmacy, Medical University of Gdańsk, 80-416 Gdańsk, Poland; 2 Department of Physical Chemistry and Physicochemistry of Polymers, Faculty of Chemistry, Nicolaus Copernicus University in Toruń, 87-100 Toruń, Poland

**Keywords:** polymer, plastic, waste, degradation, microplastic, nanoplastic, environment pollution, food safety, human health

## Abstract

Plastics are commonly used for packaging in the food industry. The most popular thermoplastic materials that have found such applications are polyethylene (PE), polypropylene (PP), poly(ethylene terephthalate) (PET), and polystyrene (PS). Unfortunately, most plastic packaging is disposable. As a consequence, significant amounts of waste are generated, entering the environment, and undergoing degradation processes. They can occur under the influence of mechanical forces, temperature, light, chemical, and biological factors. These factors can present synergistic or antagonistic effects. As a result of their action, microplastics are formed, which can undergo further fragmentation and decomposition into small-molecule compounds. During the degradation process, various additives used at the plastics’ processing stage can also be released. Both microplastics and additives can negatively affect human and animal health. Determination of the negative consequences of microplastics on the environment and health is not possible without knowing the course of degradation processes of packaging waste and their products. In this article, we present the sources of microplastics, the causes and places of their formation, the transport of such particles, the degradation of plastics most often used in the production of packaging for food storage, the factors affecting the said process, and its effects.

## 1. Introduction

Plastics constitute a group of versatile synthetic materials with numerous applications. Their ubiquity has negative consequences in the form of extensive environmental pollution [1]. It is currently stated that 60–80% of garbage is plastic [2]. Due to improper environmental policies and little public awareness or ignorance, a significant amount of waste enters the environment and causes serious problems of uncontrolled pollution [3,4]. In the European Union (EU), 80–85% of marine waste is plastic, of which 50% are single-use products. These articles and their waste can slowly decompose and generate numerous smaller pieces of debris [5]. Plastic particles between 0.1 and 5000 µm in size are referred to as microplastics (MP) [6]. Particles smaller than MP, with sizes between 1 and 100 nm, are nanoplastics (NP) [7].

**Figure 1:**
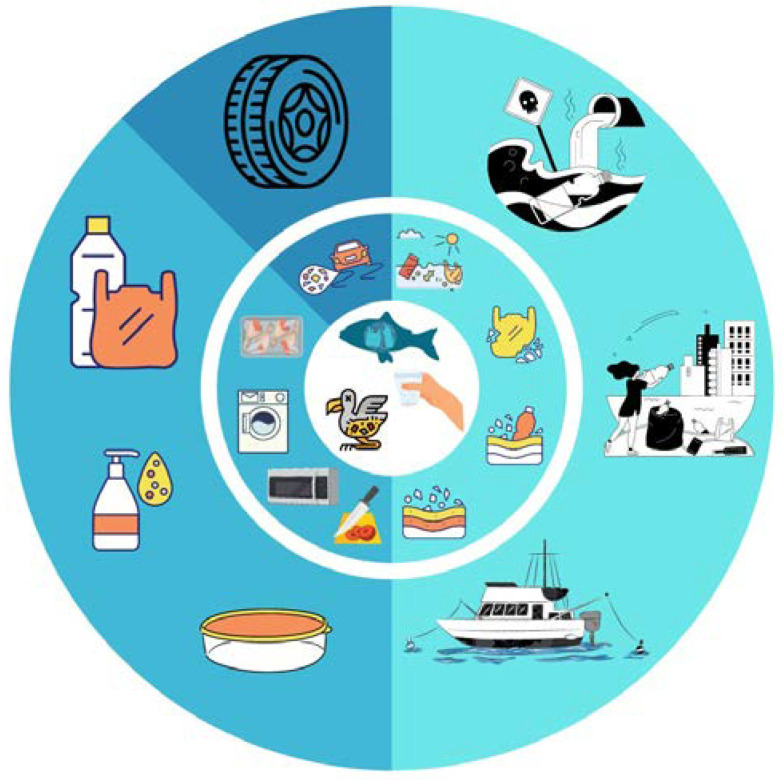
Scheme of food contamination due to MPs.

The sources of MPs, their properties, and potential harm are of widespread concern [8]. Studies have shown that such particles are present in both aquatic and terrestrial environments, posing a threat to the functioning of ecosystems [9]. Microplastics are found in soil [10], freshwater [8,11], seas and oceans [12], snow [13], wastewater [14,15], air [16], plants [17], and animal organisms [18]. The formation of MPs is a global threat [19], as they can travel as far as 6000 km [13] and enter the trophic chain [2]. Such particles can contaminate food and beverages [5,20] (Figure 1). 

**Figure 2:**
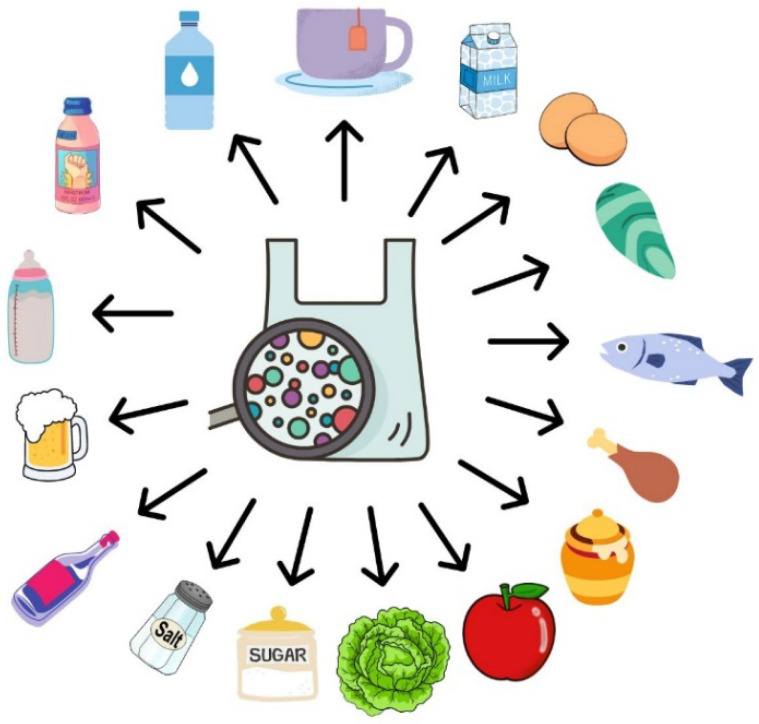
Occurrence of MPs in foods and beverages.

Microplastics have been found in fish [21], shellfish [22,23,24], poultry meat [25], eggs [26], salt [27,28,29], sugar [30], fruits [31], vegetables [32], water [33], milk [34,35], honey [35,36,37], beer [35], wine [38], tea [39,40,41], energy drinks, soft drinks [41], and infant formula [42] (Figure 2). Consumption of MPs negatively affects the digestive, respiratory, and circulatory systems [43]. They can accumulate in the body, causing inflammation. Contact with MPs is associated with the risk of oxidative stress, changes in cell division and viability, DNA damage, immune reactions, metabolic disruption, intestinal dysbiosis, and increased risk of cancer, respiratory, and neurodegenerative diseases [6,43].

Depending on their origin, MPs are divided into primary and secondary. Primary MPs are particles designed to be microscopic in size. They are used in the form in which they were produced. The degradation of plastics in the environment is considered one of the main processes contributing to the formation of secondary MPs [44]. Prolonged exposure of packaging waste to factors such as sunlight, water, temperature, and microbial action leads to its fragmentation into smaller pieces. These particles have the character of anthropogenic pollutants. The degradation of plastics is also very important with regard to forensic issues, as plastic packaging is one way to dispose of items or residues associated with criminal activity [45]. Primary and secondary MPs further degrade to NPs [46]. 

Determination of the MPs’ negative consequences on the environment, as well as animal and human health, is not possible without knowledge about the degradation of packaging waste made of plastics and their products. In this work, we paid attention to the analysis of the MPs’ sources and the reasons and circumstances for their formation. Moreover, the transport of such particles, the course of degradation processes of plastics most often used in the production of packaging for food storage, the factors affecting the said process, and its effects.

## 2. Methods

During the preparation of this article, we reviewed the literature and extracted the most relevant information regarding the locations and sources of MPs. We focused on scientific papers from ScienceDirect and Scopus databases. “Microplastic,” “food,” and “degradation” were used as the search terms in the title, keywords, and abstract. The full texts of the chosen articles were analyzed, and then the fundamental information was summarized. We devoted special attention to the papers concerning plastic degradation in the territorial and aquatic environment. The analysis of scientific articles focused on peer-reviewed papers written in English that were published as of the year 2020. Papers before 2020 were included due to their relevance to MP research. We reviewed more than 280 papers on microplastics and polymer degradation.

## 3. Food Packaging

Products made of plastics have gained popularity due to their low production costs, light weight, ease of use, and durability. It is estimated that about 39.6% of such materials are used for packaging [43]. The purpose of food packaging is protection, encapsulation, convenience, and communication with consumers. Packaging protects food from mechanical damage and microbiological and chemical contamination [47] and facilitates food storage, handling, and transportation [48]. 

Currently, there are several regulations on plastic products intended for food contact. Unfortunately, the topic of MPs is not explicitly addressed in them. They refer to polymers, plastics, and additives used at the processing stage. European Commission Regulation No. 10/2011 on plastic materials and articles intended to come into contact with food states that substances with a molecular weight of more than 1000 Da cannot be absorbed in the body, and possible health risks may be caused by unreacted monomers or said additives, which are transferred to food through migration from the material [49]. According to the legislation, the released substances must not adversely affect the organoleptic characteristics of food and exceed the permissible limits of global and specific migration. Global migration is understood as the mass of residues of all substances released from the product into food simulants. The global migration limit is equal to 10 mg per 1 dm^2^. Specific migration, on the other hand, refers only to the specific substance released from the article into the model fluids under the test conditions. The specific migration limit from plastic products was set for selected elements, i.e., Ba (up to 1 mg/kg), Co (up to 0.05 mg/kg), Cu (up to 5 mg/kg), Fe (up to 48 mg/kg), Li (up to 0.6 mg/kg), Mn (up to 0.6 mg/kg), and Zn (up to 25 mg/kg).

In addition, European Commission Regulation No. 10/2011 states that the risk assessment for substances released from packaging should include the substance itself and the degradation products arising from the intended use [49]. This statement, therefore, eliminates secondary MPs arising from the degradation of plastic packaging waste in the environment from the area of concern.

The most popular packaging for food protection and storage are containers, bottles, films, pouches, and cups [43]. They are usually made of high-density polyethylene (HDPE), low-density polyethylene (LDPE), polypropylene (PP), polyesters (such as poly(ethylene terephthalate) (PET)), and polystyrene (PS) [48,50].

Each type of polymer is characterized by different properties, and thus, they find various applications. Polyethylene (PE) is mainly dedicated to film and bags. Water bottles are made of PET, and caps are usually made of PP [5]. The release of MPs from plastic bottles and cartons was investigated. Most of the particles in water from returnable bottles were identified as PET (84%) and PP (7%), while in water from beverage cartons, other MPs, such as PE. This can be explained by the fact that the cartons are coated with PE film on the inside. In both situations, the particles are smaller than 20 μm [5]. The last of the polymers described—PS, is most often applied in the foamed form. Until recently, PS was used for disposable food packaging with heat-insulating properties. However, according to Directive 2019/904 of the European Parliament and the Council, the marketing of food and beverage containers made of expanded PS has been restricted since 2021 [51]. 

## 4. Plastics Degradation

During the storage of packaging waste made of plastics, aging occurs, that is, the gradual loss of physical and mechanical properties of the material. Degradation can happen under the influence of mechanical forces (mechanical degradation), temperature (thermal degradation), light (photodegradation), various chemicals (chemical degradation), and biological factors (biological degradation) [52]. As a result of their action, materials can fragment into macroplastics, then MPs and smaller particles (Table 1), which undergo further decomposition into small-molecule compounds, CO_2_ or CH_4_ (Table 2) [43,53]. It should be noticed that sources of MPs can be food containers made of plastic when heated in a microwave oven [54].

Unfortunately, most studies devoted to plastic degradation only address its early stages, probably because of the very long time required to achieve the goal (e.g., estimated half-lives ranging for HDPE bottles in the marine environment is 58 years) [75,76]. In the environment, however, the degradation of conventional plastics is a long-term process subjected to environmental conditions. The influence of a specific factor on the rate of degradation depends on the type of plastic, including the polymer structure, degree of cross-linking, molar mass, degree of crystallinity, and the presence of additives in the material that affect the processing and performance properties of the material (e.g., photostabilizers, heat stabilizers, plasticizers, flame retardants, nanoparticles, pigments). During aging, introduced additives can be released into food and exhibit harmful effects on organisms in contact with them (Table 3) [77]. Bisphenol A (BPA) is an example of a plasticizer [78], which release from MPs has been confirmed. A study of fishes (*Dicentrarchus labrax*, *Trachurus trachurus*, *Scomber colias*) from the Northeast Atlantic showed the presence of BPA, bisphenol B (BPB), and bisphenol E (BPE) in muscles, and BPA additionally in the liver [79]. Individuals in which MPs were detected had significantly higher concentrations of bisphenols than those whose bodies were not contaminated with plastic particles [80]. BPA (4.02 mg/L) was found to stimulate the production of reactive oxygen species, resulting in reduced biomass viability and even apoptosis [81]. Currently, BPA cannot be used in the production of baby bottles in the US. In the European Union, according to European Commission Regulation 10/2011, a specific migration limit of 0.6 mg/kg has been established for this compound [49].

### 4.1. Mechanical Degradation

Mechanical degradation refers to the breakdown of plastics due to external forces, collision, and abrasion of materials [118,119,120]. Microplastics can be introduced into food during its preparation. It has been estimated that 100–300 MPs/mm are formed on the cutting board when cuts are made during food preparation. On the other hand, in an aqueous environment, the freezing and thawing of plastics can also cause the mechanical degradation of polymers [121]. 

The effect of external forces depends on the mechanical properties of the materials [122]. Plastics with a low elongation value at break are more prone to fragmentation under external tensile forces. This leads to the tearing of polymer chains [123]. As a result of the mechanical degradation of primary and secondary MPs, smaller plastic particles (e.g., NPs) can be obtained [124].

### 4.2. Thermal Degradation

In addition to mechanical grinding, the temperature can also affect the course and efficiency of plastic degradation [125]. When enough heat is absorbed, long polymer chains can be broken, generating radicals [126]. These can react with oxygen and produce peroxides, which decompose to form free hydroxyl radicals and alkoxy radicals. The reaction can proceed spontaneously until the energy supply ceases or inert products are formed by the collision of two radicals. The temperature required for thermal degradation is related to the thermal properties of the plastics and the availability of oxygen [122]. Singh et al. concluded that the decomposition of PE occurs in one stage, between 230 and 510 °C [127]. Polypropylene has an onset degradation temperature of 286 °C [128], while PS has an onset degradation temperature of 370 °C [129]. However, the pyrolysis process of PET starts sharply and occurs at around 427–477 °C (around 90% of the process) [130].

### 4.3. Photodegradation

Photodegradation of plastics involves reactions initiated by solar radiation. As a result of the changes, plastics are gradually destroyed, with fragmentation into smaller particles and the formation of MPs [131]. As a result of UV radiation, new functional groups are formed, and the crystallinity, thermal and mechanical properties, and surface morphology of MPs change [132]. In the environment, during solar radiation, plastic waste is also affected by atmospheric oxygen, so the process is often referred to as oxidative photodegradation. Thermal oxidation of plastics occurs in conjunction with photodegradation, especially on beaches or sidewalks that are exposed directly to sunlight [133,134]. Polymers containing aromatic rings in their structure (PS and PET) were found to be more susceptible to oxidation compared to polymers formed by aliphatic chains (PE and PP) [64].

Currently, plastics in which photodegradation is an intended feature are gaining popularity. These are photodegradable materials containing sensitizers that degrade when exposed to UV light in the presence of oxygen. Polyolefins, intended for the manufacture of disposable packaging, are the largest contributor.

#### 4.3.1. Course of Plastic Degradation

Photodegradation of polymers generally involves a free radical mechanism. There are three main steps: photoinitiation, propagation, and termination. Norrish type I and II reactions produce radicals, and ketone groups, which cause cleavage of the main chain. Free radicals can react with oxygen to form superoxide radicals, which are converted into superoxide molecules. The peroxide moiety dissociates into macroalkoxy and hydroxyl radicals, which catalyze the further reaction. During the reaction, aldehydes, ketones, carboxylic acids, esters, and alcohols can be formed; moreover, chain scission and crosslinking of polymers can be observed [135]. 

Some of the photochemical reactions can be ionic or ion radical in nature. This is especially observed in the case of polymers characterized by an ionic structure.

#### 4.3.2. Effect of Various Factors on the Photodegradation of MP

The process of photodegradation of packaging waste depends on many factors. The wavelength of solar radiation, atmospheric oxygen concentration, O_3_ formation, the presence of SO_2_, NO_2_, and metallic compounds, as well as mechanical factors, are of particular importance.

##### Effect of Radiation

Photodegradation of plastics in the environment is caused by solar radiation reaching the Earth. The efficiency of the photodegradation process depends on the wavelength of light. The shorter the wavelength, the higher the radiation energy. Short-wavelength radiation, compared to long-wavelength radiation, generally induces faster and more efficient changes in the chemical structure of macromolecules and in the physical properties of the polymer. UV radiation energy of 254 nm is already sufficient to break C-C and C-H chemical bonds. While the energy of visible radiation is much lower, so it breaks only the weakest chemical bonds [136]. The impact of UV radiation on plastic packaging waste results in the embrittlement of the material and the formation of cracks and fractures at their surfaces. Song et al. found that these factors did not directly affect the fragmentation of PE and PP [137]. The formation of plastic particles required subsequent mechanical abrasion. This implies that beyond the action of UV radiation, additional physical force is required for the formation of MPs.

##### Effect of Oxygen

Plastics can undergo decomposition reactions in the presence of oxygen. This agent is involved in the oxidation cycle of the irradiated polymer, reacting with macroradicals of various types. 

Initially, photooxidation occurs in the thin surface layer of the sample, up to 100 μm [138]. The concentration of photoproducts is the greatest near the surface of the degraded plastic. For example, the effect of UV radiation on PET degradation was investigated. It was found that the process occurs at a depth of up to 20 µm [139]. 

As a result of the gradual diffusion of oxygen deep into the material, reactions can occur throughout the polymer. The efficiency of this process depends on the oxygen concentration and the properties of the polymer. Oxygen diffusion is possible in amorphous polymers. In the case of crystalline polymers, it is a limited process. Oxidation is favored by elevated temperatures and the presence of catalysts such as metals and metal ions. This results in the formation of numerous oxidation products, such as peroxides, alcohols, ketones, aldehydes, acids, peroxyacids, peresters, or y-lactones [140]. 

##### Effect of Ozone

It should be mentioned that O_3_ can be formed from O_2_ as a result of UV radiation and atmospheric discharge, which occurs naturally in low concentrations in the atmosphere. Ozone, even at low concentrations, can react with the polymer directly and attack unsaturated C=C double bonds. This reaction causes the destruction of polymer chains and the formation of carboxyl and ester groups. Ozone can also react with saturated polymers, but at a much slower rate [141].

##### Effect of Oxides

Compounds such as SO_2_ and NO_2_ are commonly found in the atmosphere. They can attack plastics directly or catalyze radical formation, which also leads to degradation. Sulfur(IV) oxide can be excited by UV radiation, forming a reactive singlet or triplet state that reacts with unsaturated C=C double bonds directly or produces O_3_ through a photochemical reaction with O_2_. Nitrogen(IV) oxide is very reactive due to the presence of odd electrons in the molecule, which can easily react with unsaturated C=C double bonds in the polymer. As with SO_2_, the photochemical reaction of NO_2_ with O_2_ also produces ozone [142].

##### Effect of Metal Compounds

Pollution of the atmosphere, soil, and water can be a source of metal cations, such as Fe, Pb, Cu, Zn, Mn, and Hg. The changes caused by their presence depend primarily on the type of inorganic compound (from which they originate) and the structure of macromolecules. They can affect the degradation process of the plastic and absorb on the surface of MPs, which exhibit high specific surface area and hydrophobicity [143,144,145,146,147]. It was found that aged MPs showed higher sorption of heavy metals than plastic particles with an undegraded surface, indicating a higher environmental and health risk of degraded particles [148].

##### Effect of Mechanical Factors

It was found that in the presence of UV radiation, plastics are more susceptible to mechanical abrasion [137]. The action of mechanical forces on plastic packaging waste causes the fragmentation and formation of surface defects on the surface of MPs, the presence of which is associated with the breakage of polymer chains. As a result of this process, free radicals are generated, which are initiators of photodegradation. At the same time, the aforementioned microdefects increase the surface area of MPs and facilitate the diffusion of atmospheric oxygen into their depths.

#### 4.3.3. Changes in the Properties of MPs

It was found that photodegradation in air causes a decrease in molecular weight, and the mechanical and physicochemical properties of plastics. Moreover, it changes the appearance and texture of the studied material [149]. As a result of UV radiation, MP particles become brittle, their surface properties switch, and roughness and porosity increase. Furthermore, the hydrophilicity and, thus, the adhesion and wettability properties change. Particles, which are colorless by nature, turn yellow under UV radiation due to the formation of sequences of conjugated double bonds of different lengths. In addition, excipients that modify the properties of the polymer can decompose when exposed to light and initiate degradation of the macromolecules.

#### 4.3.4. Photodegradation of Selected Polymers

The probability of initiated photodegradation, C-H oxidation, and chain scission depends on the structure of the polymer [150,151]. Susceptibility to photodegradation is related to the presence of UV-absorbing chromophore groups in macromolecules. Polymers containing aromatic rings or carbonyl groups in their structure are sensitive to photochemical degradation. Macromolecules that do not contain chromophore groups in their structure also undergo photodegradation. However, it is caused by the presence of structural defects or trace amounts of impurities, including catalyst residues [151,152]. Macromolecules without tertiary hydrogen groups were found to be very stable. 

A comparison of plastics with and without tertiary C-H bonds reveals that reactivity (i.e., bond dissociation energies) decreases as follows: PS > PP > PE [153]. Similar relationships were obtained by performing a study of the effect of simulated sunlight. Fragmentation initiation proceeded in the order PS (<1 year) > PP (<2 years) > LDPE (>3 years) [132]. The lowest degradability of polyolefins can be explained by the high level of hydrophobicity [154]. 

##### Polyethylene and Polypropylene

The photodegradation of PE and PP is similar. However, PP is less stable than LDPE and HDPE due to the presence of a tertiary carbon in the main chain, which is more susceptible to oxygen attack [155]. Taking into account the photochemical stability of the listed polyalkanes, they can be ranked in the following order: PP, LDPE, and HDPE. 

**Figure 3:**
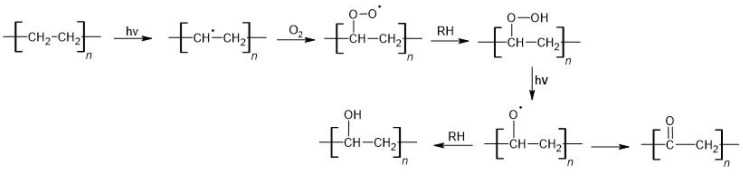
Photodegradation of PE (hν—to add energy via photons, RH—inclusions).

The formation of radicals under UV radiation in PE is made possible by the presence of various types of inclusions (RH). These contaminants are residues from unreacted reagents used during polymerization or material processing (e.g., initiators, catalysts, solvents, pigments). As a result of UV exposure, the RH decomposes into R˙ and H˙ radicals, which react with the polymer and cause the formation of radicals in the macro-chains. The following reactions involving radicals lead to random chain disruption and the formation of lower molecular weight degradation products [156,157]. The oxygen diffusion coefficient in polyalkenes is about twice as low as in their low-molecular-weight homolog. During the photooxidation of PE (with RH participation), carbonyl and hydroxyl groups are formed (Figure 3), as well as H_2_O, CH_4_, methanol, propanone, CO, and CO_2_. At the same time, conjugated double bonds can be formed. The oxidative degradation process of PE was carried out. It was found that C-H bonds oxidize and carbonyl groups are formed, which facilitates the formation of biofilms [158].

It was found that as a result of photo- and oxydegradation of PP, the morphology of the particles and their hydrophobic properties change [159], and a reduction of at least 65% volume is observed [57]. Polypropylene mainly undergoes chain breakage, depolymerization, and photo-oxidation reactions. It should be stressed that in the case of this polymer, isolated double bonds are formed.

A model study of isotactic PP films, commonly used in packaging, was conducted to simulate the process of MP formation using UV. Shredding of the tested material into sub-millimeter particles was observed in less than 48 h. This allowed an estimation of the lifetime of this type of product between 9 months and 3.2 years, depending on the place and climate in which the waste is located [19]. In another study, in a simulated beach environment, 12 months of UV exposure and 2 months of mechanical abrasion to PP and PE resulted in the formation of approximately 6084 and 20 particles, respectively [137].

##### Poly(Ethylene Terephthalate)

**Figure 4:**
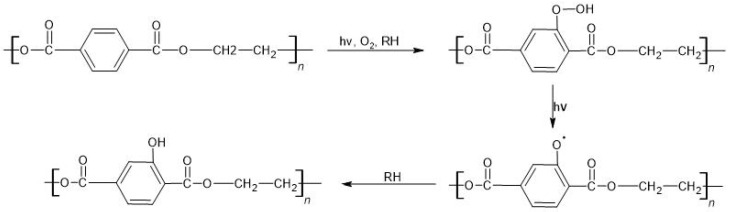
Photodegradation of PET (hν—to add energy via photons, RH—inclusions).

Poly(ethylene terephthalate) is a polymer that is resistant to environmental and biological factors [160]. However, it can be photodegraded through radical reactions. As a result of this process, PET packaging waste loses its mechanical properties; moreover, the formation of surface microcracks and color changes are observed. Poly(ethylene terephthalate) degradation is initiated by radiation with a wavelength of λ < 315 nm. Then, the alkyl and phenyl radicals created undergo reactions with oxygen, forming hydroxyl, aldehyde, and carboxyl groups at the ends of the chains. As a result of photooxidation, hydroxyl groups are also produced in aromatic rings. Hydroxyls can react with aromatic rings in the polymer backbone to make hydroxyterephthalate groups (Figure 4) [161,162]. Photodegradation of PET leads to the cleavage of the ester bond. As a consequence of this process, CO, CO_2_, terephthalic acid, anhydrides, carboxylic acids, and esters can be created [152].

##### Polystyrene

**Figure 5:**
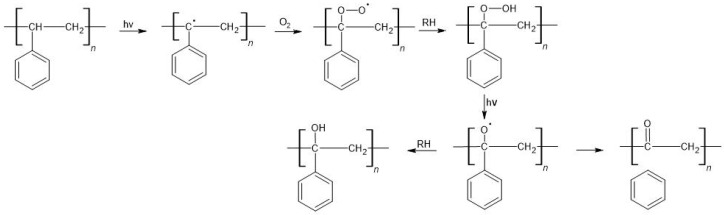
Photodegradation of PS (hν—to add energy via photons, RH—inclusions).

Polystyrene is susceptible to photodegradation due to the presence of phenyl rings, which under UV radiation (200–300 nm), become excited and form a triplet state. As a result of UV absorption, the following changes are observed: main chain breakage, hydrogen atom stripping, and phenyl ring stripping. During the degradation process, macroradicals are formed, which in the next step, undergo oxidation in the presence of atmospheric oxygen, with the formation of superoxide radicals. Subsequent reactions lead to the formation of hydroperoxide, hydroxyl, and carboxyl groups. Eventually, chain scission occurs, forming carbonyl compounds, benzene, styrene, and olefin (Figure 5) [163,164]. In summary, polystyrene MPs can be formed by photodegradation. These particles will be further decomposed. It was shown that 12-month UV exposure and 2-month mechanical treatment of expanded PS (EPS) allowed the observation of 12,152 MPs [137]. The quantities of small-molecule degradation products released during irradiation can show both an upward trend (e.g., benzene and toluene) and a downward trend (e.g., styrene and 2-propenylbenzene) [70].

### 4.4. Chemical Degradation

The most important chemical factors affecting the degradation of plastics in an aqueous environment are the pH value and salinity of the water. High concentrations of H^+^ or OH^−^ in the aqueous environment can catalyze the degradation of plastics that are susceptible to hydrolysis, such as polyamide (PA) [165]. These two factors can also affect the surface of MPs, their properties in aqueous environments, and their affinity for other contaminants. Polyethylene and PS in the form of MPs were studied by Liu et al. [166]. In the mentioned work, it was found that the presence of NaCl and CaCl_2_ increases the sorption of both diethyl phthalate (DEP) and dibutyl phthalate (DBP) [166].

### 4.5. Biological Degradation

The biological degradation of plastics is determined by organisms (e.g., bacteria, fungi, and insects) that can destroy materials physically through biting, chewing [167], or biochemical processes [122,168]. The ingested plastics can be retained in the stomach, where fragmentation will occur, subsequently releasing particles [169]. This process can be accelerated by abiotic degradation, resulting in the formation of low molecular weight degradation products and the formation of cracks and pores on the surface of the plastic [170]. The biological degradation of PP with *Bacillus* sp. strain 27 and *Rhodococcus* sp. strain 36 made it possible to conclude that this is a process dependent on the type of microorganisms. In the first case, the weight loss was found to be 4.0%, and in the second, 6.4% [59]. In terms of degradation potential, the type of polymer is also important. It was found that bacteria degraded PP more easily than PE, while fungi degraded PE more easily than PP [58]. However, among synthetic polymers, aliphatic polyesters are the most susceptible to microbial degradation. It is widely believed that the ability of microorganisms to degrade synthetic polyesters is due to their chemical similarity to natural polyhydroxybutyrate (PHB), which is the backup material of many bacterial strains. Depending on the absence or presence of ester and amide groups, plastics can be attacked by various extracellular hydrolases. It is assumed that polyesters are degraded by enzymes, such as proteases, esterases, lipases, and cutinases. 

The degradation of polymers that do not contain ester and amide groups by extracellular enzymes is a very complicated process. These polymers can be oxidized by O_2_ with hydrolase catalysis, resulting in low-molecular-weight degradation products. Laccase enzyme has played a major role in PE degradation by *Rhodococcus ruber*. The activity of laccase is improved by the presence of copper. Hydroquinone peroxidase, on the other hand, was found to be responsible for PS degradation by *Azotobacter beijerinckii* HM121 [171]. The biological degradation of plastics can also be caused by algal enzymes [172].

Degradation with the participation of extracellular enzymes breaks polymer chains with the yield of shorter-chain polymers as well as oligomers, dimers, and single molecules [173]. Ultimately, plastics can be mineralized to CO_2_ and H_2_O under aerobic conditions and to CH_4_, CO_2_, organic acids, H_2_O, and NH_4_. Degradation of plastics under anaerobic conditions is energetically disadvantageous compared to degradation under aerobic conditions, and complete mineralization can take much longer [174].

## 5. Packaging Waste Dump

Packaging waste is ubiquitous. It can be transported from land to water and from water to land [175,176,177,178]. Microplastics formed in land and water can also move between different ecosystems [179,180]. These environments are commonly thought of as independent, but in fact, they are closely interconnected [181].

### 5.1. Terrestrial Environment

Microplastic in the terrestrial environment is formed by the fragmentation of larger plastics into smaller pieces due to exposure to UV radiation, wind action, agricultural activities, oxidation processes, and chemical and biological interactions [9,180,182]. The combined effects of the aforementioned factors can accelerate the aging of MPs, manifested by changes in color, crystallinity, chemical composition, and surface properties [183]. Microplastic in the terrestrial environment affects soil quality and biota [146,184]. For example, it has been found that the presence of MPs can significantly reduce the volume of phosphates available in the soil [185].

#### 5.1.1. Sources and Transport of Microplastics in the Terrestrial Environment

Significant amounts of MPs are generated in landfills, peri-road areas, and agricultural areas [186,187]. Soil contamination can come from many sources, including compost [147], mulch film [188], greenhouse materials, irrigation tools [189], plant protection products, fertilizers [190], municipal solid waste, sewage treatment plants [191], used tires [119,164], and precipitation [192]. The presence of plastic particles in soils from China [193,194,195], Iran [196], Brazil [197], and Spain [198] was confirmed. It was found that the distribution of MPs in soils showed differences not only regionally but also in-depth [10]. The movement of MPs with groundwater can cause pollution of freshwater ecosystems, also contributing to marine pollution [199].

Plastic particles that reach the soil surface are transported to deeper layers of the soil through cultivation, infiltration, and animal activity [9,189,200]. Polyethylene beads can be transported from the soil surface down the soil profile by *Lumbricus terrestris* [200].

Polyethylene is the most commonly used polymer to study the degradation of plastics in soil. The degradation of this material was found to be increased by elevated pH and humidity. Polyethylene bags buried in soil for 2 years showed an increase in surface roughness. A nearly 5% decrease in weight was found for commercial carrier bags made of PE stored in mangrove soil over 8 weeks. This was due to the action of heterotrophic bacteria capable of producing hydrolytic enzymes [201]. In an experiment on the degradation of plastics buried in soil for 32 years, significant bleaching of LDPE film was observed, but no evidence of PS degradation was observed [202]. Thus, further studies are needed to determine the effects of individual polymers on soil properties and functions. These analyses should consider a wide range of particle sizes and shapes, as well as different types of substrates.

#### 5.1.2. Impact of Microplastic on the Terrestrial Environment

MPs-induced changes affect soil function and the soil microbial community. The presence of the described particles can directly or indirectly affect chemical processes in the soil environment and the circulation of water, nutrients, and geochemical elements. The size distribution of soil aggregates changed when MPs were present, suggesting potential changes in soil stability. It was found that the PE film increased the evaporation rate of water by creating channels for moving water [187]. Biogenic transport of MPs in the soil can lead to groundwater contamination [203,204], results in uptake by plants [205], and causes changes in root biomass. Microplastics have the potential to affect plant growth and can accumulate in plants [9]. A reduction in seed germination and shoot length of *Lolium perenne* was found after exposure to poly(lactic acid) PLA. In contrast, the biomass of *Aporrectodea rosea* was significantly reduced as a result of HDPE exposure compared to control samples [188].

In addition, MP present in the soil affects invertebrates living in this environment and can penetrate the intestinal walls of soil nematodes, causing oxidative stress and affecting gene expression [206]. It was found that the moist soil environment had a pronounced effect on the release of plasticizers [45].

### 5.2. Aquatic Environment

Aquatic ecosystems are very diverse chemically, physically, and biologically. Microplastic research in aquatic environments includes analysis of pollution from rivers and lakes [207,208,209] to seas and oceans [210,211,212,213] and the Arctic ice shelf [214]. The freshwater environment can differ from the marine environment in several aspects, including the intensity of sunlight, the physicochemical properties of the water, and its biological properties. The results of plastic degradation indicate different rates of this process depending on specific environmental conditions. Microplastic accumulates both on the water surface and in sediments [2,215]. 

#### 5.2.1. Sources and Transport of Microplastics in the Aquatic Environment

Plastic packaging waste can be transported downwind and downstream. It is believed that most MPs in the aquatic environment are formed by weathering of plastic waste [216]. This process consists of photodegradation and mechanical, chemical, and biological degradation. Plastics on the surface or in the photic zone of water can undergo photodegradation under UV radiation. It is responsible for the initial degradation of plastics floating on the surface of seawater. Mechanical degradation is caused by waves. Chemical degradation in the aquatic environment mainly involves the breakdown of chemical individuals under the influence of various compounds [52]. Plastics in water can be colonized by microorganisms that form a biofilm and break down organic matter. Biofilm formation will limit light transmission [181]. Plastics can be very persistent on the seafloor and in sediments due to UV protection and low oxygen content [185]. Biological degradation by microorganisms in the biofilm is the main cause of plastic degradation in seawater in the aphotic zone. The rate of plastic degradation can be reduced by low water temperatures [216]. As a result of degradation in the aquatic environment, weight loss, changes in the appearance and structure of plastics, and deterioration of mechanical properties will be observed [159,217,218].

The degradation of plastics in marine, river, and lake ecosystems was investigated. Poly(ethylene terephthalate) was found to be the dominant polymer in the coastal waters of Hainan Island in China (South China Sea), occurring as white-black linear or fragmented particles [12]. It was observed that PET bottles collected from the seafloor were found to remain robust for about 15 years, after which a significant decrease in native functional groups was observed [219]. However, in the case of LDPE, rapid initial decomposition within the first week, followed by little further loss of tensile strength over 4 months, was established [220]. The total PE, PP, and PS mass, with a particle size of 32–651 µm suspended in the upper 200 m of the Atlantic Ocean, was determined to be 11.6–21.1 million tons [214]. In addition, PE and PS were shown to be present in the Wei River Basin, which is located in northwest China [203]. The occurrence of PE and PP-sized MPs was confirmed in the Thames River [209]. Particles of this type were also present in the waters of another European river, the Rhine [208], and in the surface waters of the Laurentian Great Lakes in the United States [207].

#### 5.2.2. Impact of Microplastic on the Aquatic Environment

Microplastics have a very long degradation cycle. As a result, they can enter natural ecosystems and accumulate. It is estimated that the maximum degradation time of PE in the deep sea is about 292 years [221]. The phenomenon of MPs accumulation has been proven in more than 200 species of freshwater fish [222,223] and is linked to adverse effects on fish digestion [224,225], reproduction, and development. Microplastics can cause histopathological damage to the fish liver [226]. In addition, the adverse effects of PS and PP on *Danio rerio* fish embryos were evaluated. A reduction in body length and heart rate was observed [227].

## 6. Biodegradable Plastics

Biodegradable plastics constitute interesting types of materials. This group includes natural polymers, polymers obtained by modification of natural polymers, and polymers produced by various chemical synthesis methods and biotechnological processes. The degradation time depends on the conditions and the types of polymers. They can, in a relatively short time, be degraded to H_2_O, CO_2_, and CH_4_ [228]. The rate of degradation of biodegradable polymers depends on the environment in which they are stored [228]. For example, it was found that poly(butylene succinate-co-butylene adipate) (PBSA) degrades at 37 °C, with the generation of CO_2_ for 40 days [229]. The complete degradation of PLA can be achieved at composting temperatures of 60 °C or higher [230,231], but it does not degrade in seawater [223]. Itävaara et al. found that 90% mineralization degree of PLA was reached at 60 °C within 120 days [232]. 

However, laboratory-defined degradation conditions are virtually unattainable under natural ones. The degradation of such materials will take longer in the environment compared to the degradation time in the laboratory. Some biodegradable plastics will fragment and slowly accumulate in the environment in the form of MPs and NPs [233]. 

Microplastic derived from biodegradable plastics may show similar or even higher harmfulness to organisms compared to conventional materials. For example, PLA in the form of MP has higher toxicity to *Chlorella vulgaris* compared to PA and PE [234]. Moreover, it shows a higher adsorption capacity of tetracycline (TC) and ciprofloxacin (CIP) compared to PVC [235].

## 7. Implication of MPs Contamination on Human Health and Toxicological Studies

People’s exposure to MPs passes through ingestion, inhalation, dermal contact, and the mucous membrane of the eye surface [6,236]. The most common reason for exposure to MP is the gastrointestinal tract. Senathirajah et al. (2021) estimated the global average ingestion of MPs in the range from 0.1 to 5 g weekly [237]. While Cox et al. (2019) defined that annual MPs consumption ranges between 39,000 and 52,000 particles, increasing to 121,000 when inhalation is considered [238]. The significant source of MPs food contamination is plastic packaging waste. However, other individual factors, e.g., disposable crockery and cookware, constitute the source of MPs in food [239,240,241,242] (Table 4).

Information regarding MP is scattered. This makes the proper interpretation more difficult, and it is particularly troubling in view of the influence of this type of particle on organisms [248,249,250,251,252,253,254,255,256,257,258]. Microplastic introduced to an organism locates itself in the gastrointestinal tract and influences physiological processes. The presence of MP in the gastrointestinal tract has been proven by studying human feces [259,260,261]. Currently, it is considered that the sole measure of human exposure assessment on MPs is the discovery and quantification of plastic particles in these samples. The optical method is used to estimate MPs in human feces through involuntary ingestion [262]. Based on the conducted analysis, it has been determined that the concentration of PET in the feces of infants is ten times higher than in samples taken from adults [261]. Microplastic has also been detected in meconium samples. This fact, while disconcerting, is no surprise at all within the context of research that has confirmed the presence of MPs such as PE, PP, polyurethane (PU), PS, PVC, and poly(butylene succinate) (PBS) in the human placenta [263,264].

**Figure 6:**
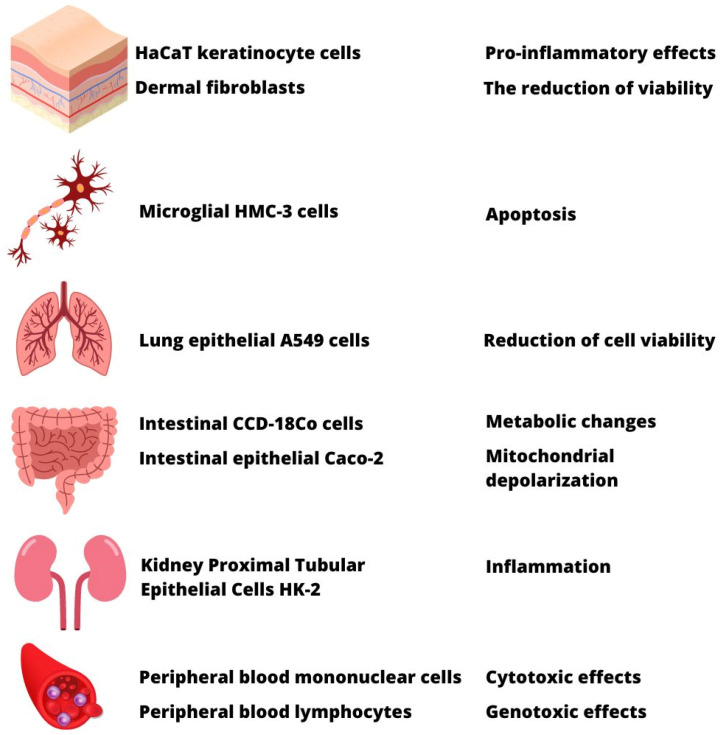
Toxicological studies and the implication of MPs contamination on human health.

While the vast literature shows that MP accumulates in living organisms [6,255,257,265], information regarding the harmfulness of this type of particle for people is limited (Figure 6). Since mice constitute a common mammal model, one should very precisely follow research regarding the influence of this pollution on the functioning of their organisms. It allows for an extrapolation of the results into humans with a perspective to assess health risks. Research on mice proved that such particles might cross the brain-blood barrier [257]. This information is particularly alarming because MPs presence was confirmed in human blood [255].

The impact of MPs on human health depends on many factors [266]. The size, shape, and chemical composition of plastic particles are the most significant ones [262]. However, personal characteristics are also important, including age, organism size, demographic features, and lifestyle [267]. 

Microplastics can induce oxidative stress in the cells. The immune system recognizes MP as an enemy, first reacting violently, the increase of antioxidation defense is observed, then the organism is weakened [268]. Thus, performing long-term research is important to discover the true effects of MPs on human health and the environment [269].

## 8. Identification Methods of MPs

**Figure 7:**
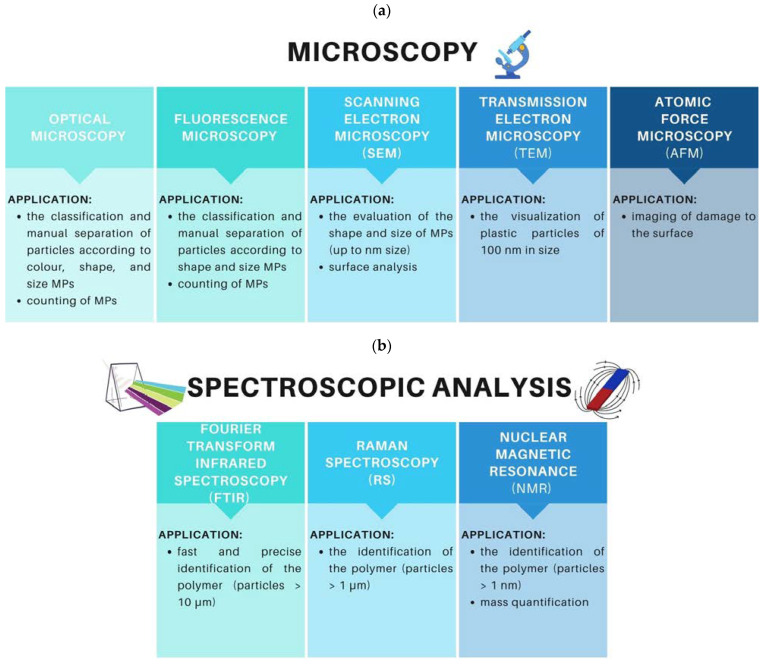
Identification methods of MPs (**a**) microscopy (**b**) spectroscopic analysis.

MPs are analyzed through several stages, such as separation, identification, visualization, and quantification. Techniques used to characterize MPs are mainly microscopic (optical microscopy, fluorescence microscopy, Scanning Electron Microscopy—SEM, Transmission Electron Microscopy—TEM, and Atomic Force Microscopy—AFM) and spectroscopic (Fourier Transform Infrared Spectroscopy—FT-IR, Raman Spectroscopy—RS, Nuclear Magnetic Resonance—NMR) methods [270,271,272,273,274,275,276,277,278,279,280,281,282,283,284,285] (Figure 7). They are mostly used to identify the polymeric composition of MPs, analysis of the shape, color, and size of the particles, as well as their quantity in test samples.

The combination of FTIR spectroscopy and optical microscopy (µ-FTIR) as well as Raman spectroscopy and microscopy (µ-Raman), are the two popular techniques used to identify MPs due to their sensitivity to small particles and accuracy in characterization. µ-FTIR methods are time-consuming, but sample preparation is relatively simple, making it a useful tool for the identification of particles up to 10 µm [274,276,277,278,279,280]. However, µ-Raman spectroscopy constitutes a reliable approach for analyzing particles as small as 1 µm [273]. With the application of these two methods, it is possible to characterize MPs accurately and reliably, making them invaluable tools for the analysis of plastic particles. These techniques are used to analyze environmental samples [272,273,274,275,276,277,278,279,280,281,282] (Table 5).

## 9. Conclusions

Plastic packaging waste is subjected to abiotic and biotic degradation processes. Factors affecting the said process can have synergistic or antagonistic effects. However, an analysis of the cases described in the scientific literature allows us to conclude that they are mostly convergent processes. They cause oxidation and disruption of polymer chains and lead to fragmentation, with the formation of MPs. The degradation of plastic packaging waste is a long and complex process that depends on the material’s composition, physicochemical and mechanical properties, and its interaction with the environment. It is believed that the most important variables associated with plastic degradation are visible light and the presence of NO_2_ and O_3_. The changes in properties observed due to photodegradation and thermal and chemical degradation affect mechanical properties, particularly their elongation at break and tensile modulus. Degradation of the plastic in the environment is able to reduce the values of elongation at break, which decreases the value of external forces required for fragmentation. Moreover, it facilitates the fragmentation and formation of MPs.

Microplastic is ubiquitous. It is found in both terrestrial and aquatic environments. These particles accumulate in natural ecosystems and adversely impact some animals and plants, as well as affect soil functions. Microplastics can take up to 292 years to degrade in the deep sea. They can have adverse effects on the aquatic environment and especially on fish digestion, reproduction, and development. The particles of plastics cause changes in the size and distribution of soil aggregates, increase the evaporation rate of water and lead to groundwater contamination. Moist soils can cause them to release plasticizers. In addition, MPs affect plant growth and reduce seed germination, shoot length, and root biomass. They penetrate the intestinal walls of soil nematodes, resulting in oxidative stress and influencing gene expression.

Research results published to date show the prevalence of MP in food and beverage products. However, the evaluation of food contamination by plastic particles is still at a very early stage. In order to conduct it effectively, gaps in analytical methodologies and toxicity studies of such particles must be identified and eliminated. However, a correct analysis of the harmfulness of MP will not be possible without understanding the causes and products of plastic degradation.

Over the past 10 years, the number of scientific and popular science publications on MPs has increased dramatically. The topic of MPs is gaining increasing attention from scientists, the public, policymakers, and regulators. It is a problem already recognized internationally. Its solution may lie in conducting appropriate environmental education, introducing more efficient packaging waste management, and searching for, developing, and implementing effective and economically viable technologies for removing MPs from our environment.

## Figures and Tables

**Table 1:** Microplastics degradation effects.

Polymer Type	Degradation Method	Effect	References
PE	Photodegradation	Oxygen functional groups on the surface; the increase of specific surface area	[55]
	Chemical degradation (prothioconazole)	Cracks	[56]
PP	Photodegradation	Reduction in microplastic particle volume	[57]
	Biological degradation (*Serratia marcescens* and *Enterobacter* spp.)	Surface changes (microcracks and corrugations)	[58]
	Biological degradation (*Rhodococcus* sp. And *Bacillus* sp.)	The reduction of the polymer mass; structural and morphological changes in PP	[59]
PE, PP	Biological degradation (*Spirulina* sp.)	Changes of functional groups; a decrease in carbon in PE and PP	[60]
PS	Biological degradation (*Bacillus cereus* CH6)	The surface morphology changes	[61]
PET	Chemical and thermal degradation	Changes in surface morphology, crystallinity, and carbonyl index	[62]
PS, PET	Biological degradation (*Bacillus* sp.)	Structural and surface changes; weight loss; a decrease in the carbon content	[63]
PE, PP, PS, PET	Chemical degradation (Fenton’s reagent)	Wrinkles, voids, and holes on the surface; oxygen functional groups on the surface; increased hydrophilicity and acidity of the surface; reduced MP size	[64]

**Table 2:** Decomposition of plastics into small-molecule compounds.

Polymer Type	Degradation Method	Degradation Products	References
PE	Thermal degradation	H_2_, CH_4_, C_2_H_4_, and C_3_H_6_	[65]
	Photodegradation	CO_2_, H_2_O	[66]
	Photodegradation	C_2_H_6_, CO_2_, H_2_O, and formaldehyde	[67]
	Chemical degradation (O_3_ and H_2_O_2_)	3-pentanol, 3-pentanone	[68]
PP	Photodegradation	Formaldehyde, acetaldehyde, 2-propynyl, hydroxypropyl, acetone, 2-propenyl, butanal, 4-pentyn-1-olate, 4-pentyn-1-olate, (2-ethoxyethyl)oxonium, and acetylacetonate	[57]
PS	Biological degradation (the microbially driven Fenton reaction)	2-isopropyl-5-methyl-1-heptanol, nonahexacontanoic acid	[69]
	Photodegradation	Benzene, toluene, phenol, styrene, and 2-propenylbenzene	[70]
	Photodegradation	Acrolein, benzene, propanal, methyl vinyl ketone, and methyl propenyl ketone	[71]
PET	Biological degradation (*Rhococcus* sp. *SSM1*)	Monomer—terephthalic acid (TPA)	[72]
	Biological degradation (petase)	Mono-(2-hydroxyethyl) terephthalate, bis-(2-hydroxyethyl) terephthalate and ethylene glycol	[73]
PE, PP, PET, PS	Chemical degradation (Fenton oxidation)	CO_2_	[74]

**Table 3:** Health effects of additives used in plastics processing.

Additive Type	Example of the Chemical Compound	Health Effects	References
Plasticizers	Phthalates [di(2-ethylhexyl)phthalate (DEHP), diethyl phthalate (DEP), and dibutyl phthalate (DBP)]	Increased oxidative stress and inflammation: - inhibition of human salivary aldehyde dehydrogenase (hsALDH) - impact on peroxisome proliferator-activated receptor-α. Disruption in the endocrine system: - phthalates can affect the adrenal cortex (H295R cells) and cause significant disturbance of steroid hormones synthesis - decreased in testosterone and increased in 17β-estradiol. Impairment of the reproductive system: - reduction in ovulatory follicles - oocytes with poor maturation - DEHP decreased testicular function in rats - the perinatal DBP and DEP exposure may show significant growth retardations and also affect brain development and emotions like attention problems, anxiety, and depression.	[81,82,83,84,85,86]
	Acetyl tributyl citrate (ATBC)	Risk of interaction with drugs: - ATBC-induced cytochrome P4503A4. Reproductive system disorders: - ATBC decreased the number of primordial, primary, and secondary follicles present in the mice’s ovary.	[87,88]
	Poly(ethylene glycol) (PEG)	Allergy: - PEG is a risk factor for IgE-mediated anaphylaxis.	[89,90]
	Bisphenol A	Endocrine effects: - increased α-chymotrypsin activity - increased oxidative stress (BPA produces reactive oxygen species (ROS)). Reproductive system disorders: - BPA decreased viability of bovine theca cells in vitro - higher BPA exposure was associated with lower semen quality in Chinese men and endometriosis in women. Neurodevelopmental disorder: - creatinine-adjusted BPA levels were associated with a 3.3–3.6% increase in attention-deficit hyperactivity symptoms (ADHD) rating scale IV. Renal function disorders: - BPA exposure may negatively impact on kidney function and structure.	[91,92,93,94,95,96,97,98]
Antioxidants	Arylamines	Pro-cancer activity: - exposure to arylamines is associated with a higher risk of bladder cancer (mainly *p*-phenylenediamine). Autoimmune diseases: - arylamines are reported to cause lupus-inducibility.	[99,100]
Light stabilizers and ultraviolet (UV) absorbents	Hindered amines light stabilizers (e.g., bis(2,2,6,6-tetramethyl-4-piperidyl) sebacate)	Cytotoxic effect: - decreased viability and activity in epithelial cells.	[101]
	Benzotriazole UV stabilizers [e.g., UV-328 (2-(2H-benzotriazol-2-yl)-4,6-di-tert-pentylphenol)]	Inflammation: - their metabolites in human blood increased oxidative stress. Gene expression profiling: - UV-320 was a strong Peroxisome Proliferator-Activated Receptor α (PPARα) agonist in mice.	[102,103]
Heat stabilizers	Vinyl chloride	Liver diseases: - exposure to vinyl chloride was associated with cirrhosis and hepatocellular carcinoma.	[104,105,106,107]
Flame retardants	Short, medium, and long chlorinated paraffins (SCCP/MCCP/LCCP)	Cytotoxic effect: - 10–15% lower relative cells viability - SCCPS caused cell membrane damage.	[108,109]
	Tris(1,3-dichloro-2-propyl) phosphate (TDCPP)	Cytotoxic effect: - TDCPP inhibited cell growth, decreased cell viability, and increased cell toxicity in vitro.	[110,111]
	Sb_2_O_3_	Pro-cancer activity: - increased the cancer risks for inhalation exposure - induced DNA damage. Inflammation: - increased oxidative stress.	[112,113]
	Polybrominated diphenyl (PBB) and polybrominated diphenyl ethers (PBDES)	Pro-cancer activity: - increased risk of thyroid cancer. Metabolic disorders: - diabetes and metabolic syndrome.	[114,115]
Pigments	TiO_2_	Inflammation: - increased ROS production. Microbiota dysfunctions: - variations in microbiota abundance, gut dysfunctions, and reduction in short-chain fatty acids (SCFAS) levels.	[116,117]

**Table 4:** The origin of MPs from various individual sources.

Sources of MPs	Quantity of MPs	Polymer Types	References
Effluents	50–86 MPs/dm^3^	PE, PP, and PS	[243]
	840–3116 μg/dm^3^	PE, PP, PET, PVC, and PMMA	[244]
	1.2–23.1 μg/ dm^3^	PET	[245]
Bees	–	Polyester, PE, PVC, PU, epoxy resin, PAN, POM, PP, PS, PSU, PTFE, and PA	[246]
Boat ropes	11–822 MPs/m	PP, polysteel (a blend of PE and PP)	[247]
Take-out food containers	3–29 MPs/container	PS, polyester, rayon, acrylic, nylon, PE, PP, and PET (depending on the container type)	[239]
Disposable cups (pe-coated paper cups)	675–5984 MPs/dm^3^	PE	[240]
Disposable cups (PP cups)	781–4951 MPs/dm^3^	PP	
Disposable cups (PS cups)	838–5215 MPs/dm^3^	PS	
Plastic bottles for children	16.2 million MPs/dm^3^	PP (bottle material)	[241]
Cutting board	100–300 MPs/mm per cut	–	[242]

poly(vinyl chloride)—PVC; poly(methyl methacrylate)—PMMA; polyurethane—PU; polyacrylonitrile—PAN; polyoxymethylene—POM; polysulfone—PSU; polytetrafluoroethylene—PTFE; polyamide—PA.

**Table 5:** Occurrence, analysis, and abundance of MPs in the environment.

Occurrence of MPs	Methods of MPs Analysis	Abundance of MPs	References
The Chukchi Sea, western Arctic Ocean	FTIR	0–18,815 MPs/km^2^; 0–445 g/km^2^	[272]
The open Baltic Sea	Optical microscope, FTIR	79 ± 18 MPs/m^3^	[273]
Surface waters of the Kattegat/Skagerrak, Denmark	μ-FTIR	11–87 MPs/m^3^	[274]
Estuarine surface water in Mauritius	Optical microscope, FTIR	249–412 MPs/dm^3^	[275]
The water columns of catchments in Kamniška Bistrica, Slovenia	Optical microscope, FTIR	59 ± 16 MPs/m^3^	[276]
The water columns of catchments in Ljubljanica, Slovenia	Optical microscope, FTIR, μ-FTIR	31 ± 14 MPs/m^3^	
Groundwater in the Haean Basin of Korea	μ-FTIR	0.02–3.48 MPs/dm^3^	[277]
Estuarine sediments in Mauritius	Optical microscope, FTIR	74–235 MPs/kg	[275]
The sediments of catchments in Kamniška Bistrica, Slovenia	Optical microscope, FTIR	22 ± 20 MPs/kg	[276]
The sediments of catchments in Ljubljanica, Slovenia	Optical microscope, FTIR, μ-FTIR	23 ± 25 MPs/kg	
The sediments of the Weser River catchment, Germany	μ-FTIR	99 ± 85 MPs/m^2^	[278]
The atmosphere of the Northwestern Pacific Ocean	Optical microscope, μ-FTIR	0.0046–0.064 MPs/m^3^	[279]
Air in the Gdańsk harbour	Optical microscope, µ-Raman	161 ± 75 MPs/m^3^	[273]
Air of Baltic Sea	Optical microscope, µ-Raman	24 ± 9 MPs/m^3^	
Air of the Gotland Island	Optical microscope, µ-Raman	45 ± 20 MPs/m^3^	
Air in the Weser River catchment, Germany	RS	91 ± 47 MPs/m^3^	[278]
Soil of the green park in Coimbra, Portugal	Optical microscope, μ-FTIR	158,000 MPs/kg	[280]
Soil of the landfill in Coimbra, Portugal	Optical microscope, μ-FTIR	150,000 MPs/kg	
Soil of industrial area in Coimbra, Portugal	Optical microscope, μ-FTIR	127,000 MPs/kg	
Soil of dump in Coimbra, Portugal	Optical microscope, μ-FTIR	126,000 MPs/kg	
Soil of the forest in Coimbra, Portugal	Optical microscope, μ-FTIR	55,000 MPs/kg	
Soil of Bhopal, India	Optical microscope, FTIR	2.5 ± 0.71–180 ± 13.44 MPs/kg	[281]

## Data Availability

Not applicable.
